# Unidirectional Optomotor Responses and Eye Dominance in Two Species of Crabs

**DOI:** 10.3389/fphys.2019.00586

**Published:** 2019-05-16

**Authors:** Yair Barnatan, Daniel Tomsic, Julieta Sztarker

**Affiliations:** ^1^ Instituto de Fisiología, Biología Molecular y Neurociencias (IFIBYNE) CONICET, Universidad de Buenos Aires, Buenos Aires, Argentina; ^2^ Departamento de Fisiología, Biología Molecular y Celular Dr. Héctor Maldonado, Facultad de Ciencias Exactas y Naturales, Universidad de Buenos Aires, Buenos Aires, Argentina

**Keywords:** unidirectional optomotor response, monocular vision, optic flow, semiterrestrial crabs, lateralization, eye dominance

## Abstract

Animals, from invertebrates to humans, stabilize the panoramic optic flow through compensatory movements of the eyes, the head or the whole body, a behavior known as optomotor response (OR). The same optic flow moved clockwise or anticlockwise elicits equivalent compensatory right or left turning movements, respectively. However, if stimulated monocularly, many animals show a unique effective direction of motion, i.e., a unidirectional OR. This phenomenon has been reported in various species from mammals to birds, reptiles, and amphibious, but among invertebrates, it has only been tested in flies, where the directional sensitivity is opposite to that found in vertebrates. Although OR has been extensively investigated in crabs, directional sensitivity has never been analyzed. Here, we present results of behavioral experiments aimed at exploring the directional sensitivity of the OR in two crab species belonging to different families: the varunid mud crab *Neohelice granulata* and the ocypode fiddler crab *Uca uruguayensis*. By using different conditions of visual perception (binocular, left or right monocular) and direction of flow field motion (clockwise, anticlockwise), we found in both species that in monocular conditions, OR is effectively displayed only with progressive (front-to-back) motion stimulation. Binocularly elicited responses were directional insensitive and significantly weaker than monocular responses. These results are coincident with those described in flies and suggest a commonality in the circuit underlying this behavior among arthropods. Additionally, we found the existence of a remarkable eye dominance for the OR, which is associated to the size of the larger claw. This is more evident in the fiddler crab where the difference between the two claws is huge.

## Introduction

When an animal, regardless of being a fly, a mouse, a lizard or a human, rotates (or in the laboratory is exposed to the rotation of the visual panorama) its vision tends to blur. To stabilize the image in the retina, animals perform compensatory movements with the eyes, the head or the whole body following the direction of the optic flow. This behavior is known as the optomotor response (OR). Animals stimulated with a pattern of high-contrast vertical strips moving clockwise (CW) or anticlockwise (ACW) perform OR in the corresponding direction with similar strength, but only if they are seeing the stimulus with the two eyes. When this experiment is repeated occluding one eye, i.e., in a monocular condition, many animals show a marked directional preference. This also happens when presenting the stimulus over one lateral visual field in a position that can only be seen by one eye in animals, like flies, fishes, etc., that possess a small field of binocular superposition. This phenomenon is referred to as unidirectional optomotor response and has been reported in various species from mammals to birds, reptiles, amphibious, fishes, and flies (Mowrer, 1936; Fukuda, 1959; Tauber and Atkin, 1968; Collewijn, 1969; Easter, 1972; Jardon and Bonaventure, 1995; Duistermars et al., 2012). In all reported cases using vertebrates, the preferred direction of stimulus motion is from the uncovered eye toward the covered eye, i.e., back-to-front (BTF) or regressive direction as seen by the viewing eye (Mowrer, 1936; Fukuda, 1959; Tauber and Atkin, 1968; Collewijn, 1969; Easter, 1972; Jardon and Bonaventure, 1995). In contrast, the few reports addressing this issue in invertebrates (only flies) show that the front-to-back (FTB) or progressive direction of motion induces a stronger optomotor response than the regressive direction (e.g., using monocular stimulation in *Drosophila melanogaster*: Duistermars et al., 2012; using monocular occlusion in *Musca domestica*: Wehrhahn and Hausen, 1980). Unlike all reported vertebrates, flies do not move their eyes when performing OR but compensate by neck movements. Crabs’ optomotor responses, on the other hand, are mediated both by eye movements (similar to those observed in vertebrates) and by body rotations. Compensatory eye movements consist of a sequence where each cycle is composed of a slow tracking phase to stabilize the image in the retina and reduce the blur, followed by a saccadic movement that restores the eye to its initial position to start a new cycle (e.g., Horridge and Burrows, 1968). In crabs, there is a strong coupling between the movement of both eyes for the OR, as probed by the fact that compensatory eye movements can be driven in a blind eye by visually stimulating the opposite eye (Horridge and Sandeman, 1964). If the eyes became immobilized, as sometimes occurs in natural condition by the presence of barnacles fixed onto the carapace around the eyes, compensations are channeled through body rotations. This response is easy to evoke and measure in the laboratory by fixing the eyestalks and measuring the rotations of the whole body (Tomsic and Maldonado, 1990). Optomotor responses have been studied in a wide variety of invertebrates including insects and crustaceans (Miller et al., 2002; Honkanen et al., 2014; Windsor et al., 2014; Caves et al., 2016; Nityananda et al., 2017). Crabs in particular have been used extensively to study OR with special focus in understanding the control system of the slow and rapid phases of the compensatory eye movements and the interactions between the two eyes. Diverse approaches were used including fixing and/or blinding one eye (in open- or closed-loop conditions), dividing the field of view of the two eyes and presenting incoherent stimuli of diverse nature in the two sides (e.g., Horridge and Sandeman, 1964; Horridge, 1965; Horridge and Burrows, 1968; Barnes and Horridge, 1969; Barnes and Barnes, 1990; Tomsic and Maldonado, 1990; Kern et al., 1993; Johnson et al., 2002). However, to our knowledge, no systematic studies have been performed to investigate whether there is a preferred direction of OR in monocularly seeing crabs.

The semiterrestrial grapsid crab *Neohelice granulata* proved to be a good model system for neuroscience research. In particular, in the last years, we have produced abundant information about how the optic neuropils are composed and organized in *Neohelice* (Sztarker et al., 2005, 2009; Medan et al., 2007, 2015; Berón de Astrada et al., 2009; De Astrada et al., 2011; Bengochea and Berón de Astrada, 2014; Sztarker and Tomsic, 2014; Bengochea et al., 2018; Scarano et al., 2018). We have gained knowledge about how visual information (of single objects) coming from the two eyes is processed, and we learnt that information from the two eyes is first combined at the level of the lobula (Sztarker and Tomsic, 2004; Scarano et al., 2018). On the other hand, we know little about the processing of the panoramic optic flow that elicits OR in the nervous system of crabs (but see Sandeman et al., 1975; Bengochea et al., 2018).

In *Neohelice*, each eyestalk is composed by about 9,000 ommatidia distributed around the eye, conferring a field of vision of about 360° (Astrada et al., 2012). In consequence, stimuli presented at different parts of the visual field are simultaneously seen by the two eyes. The resolution of the eyes, however, is not uniform. Each eye possesses areas of maximal acuity: in the vertical plane, there is a band of high vertical resolution at the eye equator and, in the horizontal plane, the acuity of the eye peaks toward the lateral part (Astrada et al., 2012; Medan et al., 2015). This could produce differential sensitivity to optokinetic stimuli when presented in different parts of the visual field as was seen in other crabs (e.g., Nalbach and Nalbach, 1987).

Here, we tested OR under monocular and binocular conditions stimulating *N. granulata* crabs in the two possible directions of horizontal optic flow. Animals displayed unidirectional OR under monocular conditions with a FTB preferred direction. Studies were then replicated using a species from a different family (*Ocypodidae*), the fiddler crab *Uca uruguayensis* where similar unidirectional OR was also found. Interestingly, in fiddler crabs, and to a lesser extent in *Neohelice*, the unidirectional OR response was conditional to the eye ipsilateral to the major claw.

## Materials and Methods

### Animals

Adult male *N. granulata* (*Varunidae* family) measuring 2.7–3.0 cm along the carapace and male fiddler crabs *U. uruguayensis* (*Ocypodidae* family) with the major claw measuring approximately 1 cm (left-clawed and right-clawed balanced) were collected in narrow coastal inlets of San Clemente del Tuyú, Buenos Aires, Argentina. Only animals with no obvious ongoing chelae regeneration were used.

Animals were maintained at the laboratory in plastic tanks (19 cm × 45 cm × 32 cm, up to 20 individuals per tank) filled to 2 cm depth using artificial marine water (salinity = 11–15%, pH = 7.4–7.6; Marinemix, Baltimore, USA). All animals were kept at 20–26°C and under natural light. All experiments were carried between 10 and 20 h, during the whole year and within the first 2 weeks after the animal’s arrival to the laboratory. Animals were not fed during their stay in the laboratory.

### Visual Stimulation

**Figure 1:**
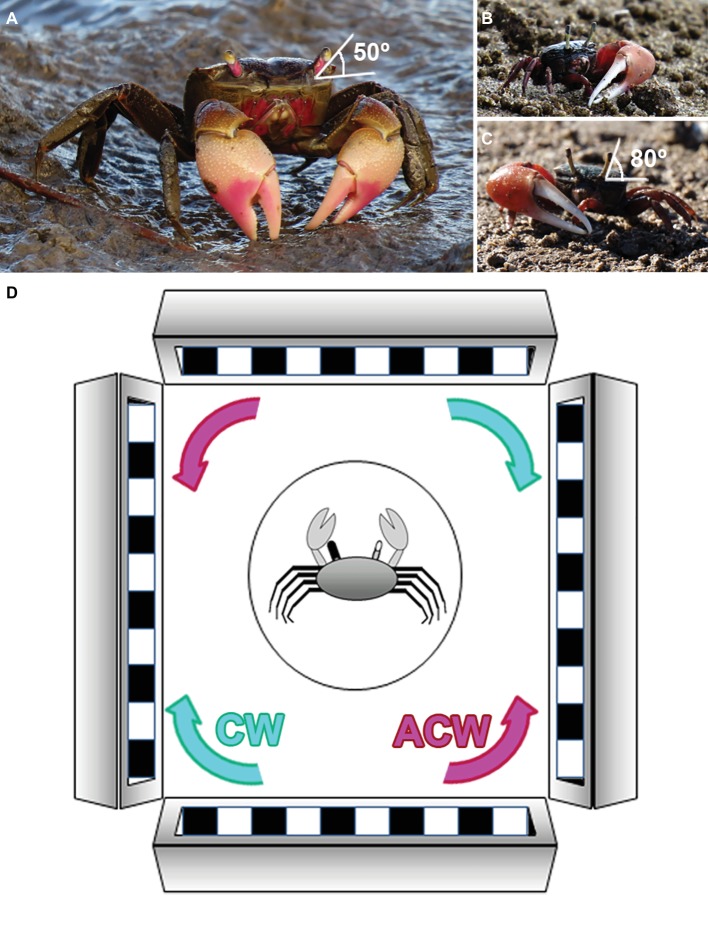
Experimental species and set up. **(A)** The varunid crab *N. granulata*. The eyestalks in this species are rather separated. Their typical seeing position is about 50° from the horizontal plane. **(B,C)** Representatives of fiddler crabs *U. uruguayensis* (*Ocypodidae* family) with the major claw on the left **(B)** or the right side **(C)**. The eyestalks are long and close together, and their typical seeing position is about 80° from the horizontal plane. **(D)** Experimental setup. We used a virtual horizontal optic flow presented coherently in an array of four flat screens fully surrounding the animal. The stimulus direction could be clockwise (CW, cyan) or anticlockwise (ACW, magenta). Crabs had their eyestalks fixed in their typical seeing position. For measuring the OR, they were individually placed inside a cylindrical glass flask (circle in the drawing) located in the center of the screen array. We used a rubber or aluminum cup to reversely cover and occlude vision of one eye (represented in this scheme as a black eyestalk). Each individual was videotaped from above to quantify the amount of turns displayed in response to the optic flow stimulation.

To trigger optomotor responses (OR), we used a virtual visual grating stimulus presented coherently in an array of four flat screens fully surrounding the animal (19-in. CRT Samsung SyncMaster, 800 × 600 pi resolution) (Figure 1). A fifth monitor located 20 cm above the animal provided a homogeneous white ceiling. Anti-glare shields in front of each screen prevented reflections. The grating pattern consisted of black and white vertical bars (2.11 cm wide, each subtending 6.3° to the center of the array, spatial frequency: 0.035 cycles/degrees) moved at 1 RPM. Each presentation of the visual stimulus lasted 3 min. Monitors had a refreshing frequency of 60 Hz, which is above the flicker fusion frequency of crabs [about 50 Hz, (Layne et al., 1997)]. The response gains obtained for *Neohelice* in this virtual setup were similar to those evoked in previous experiments using a rotating optomotor cylinder (approximately 0.4 for 1 RPM; Pérez Sáez, 2003). Therefore, the small fields occupied by the static border of the monitors do not seem to dampen the responses. These response values were also in agreement with previous accounts in other crabs (e.g., Barnes and Barnes, 1990).

### Experimental Procedure

In crabs, the movements of both eyestalks intended for compensating optic flow are highly coupled (Nalbach et al., 1993) and they have little or no role in object tracking (Barnes and Nalbach, 1993). If eyestalks motion is prevented under experimental conditions, OR is performed as whole-body compensatory movements (rotations) that are easily measured. To achieve this, animals were firmly held in an adjustable clamp, and their eyestalks were immobilized to the carapace using cyanoacrylate (loctite super glue). Care was taken to fix their eyes at their normal seeing position: *N. granulata*, 50° from the horizontal line (De Astrada et al., 2011); *U. uruguayensis*, 80° from the horizontal line (see Figures 1A–C). After eye fixation, animals were kept isolated in glass containers and they were tested between 6 and 8 days after eye immobilization. Rational behind the selection of this interval between fixation and evaluation arises from a series of pilot experiments aimed at evaluating the moment in which OR values were higher and more stable after eye immobilization. Animals were evaluated in each of the six following conditions: binocular clockwise (B-CW), binocular anticlockwise (B-ACW), right eye monocular clockwise (R-CW), right eye monocular anticlockwise (R-ACW), left eye monocular clockwise (L-CW), and left eye monocular anticlockwise (L-ACW). These conditions were presented in a pseudorandom order among different animals. Monocular conditions were achieved right before the corresponding test by occluding one eye with a removable rubber cap or a cap made of aluminum foil, allowing such condition to be reversible and painless. Efficacy of the blinding method was previously tested by occluding both eyes and stimulating with both CW and ACW motion, obtaining no OR at all (*n* = 9). On the test day, animals were placed in the visual stimulation device and were left to adapt to the new environment for 5 min, after which the experiment started.

### Behavior Quantification and Data Analysis

*N. granulata* crabs were individually placed inside a cylindrical glass flask (diameter = 8 cm, height = 16 cm) with 1 cm artificial marine water depth for avoiding hydric stress (Figure 1). A transparent lid positioned 2 cm above the crab prevented its attempts for climbing the wall of the cylinder. *U. uruguayensis* crabs were evaluated in the same way but using a proportionally smaller cylindrical glass flask (diameter = 6 cm, height = 8.5 cm).

Animal’s turns (OR) were assessed by filming them from above (Sony Cybershot G DSC-HX5) and analyzed offline by quantifying the number of quarters of body turns (90° rotation) performed around its yaw axis during 3 min of continuous panoramic motion stimulation. Positive values represent rotations performed in the same direction of the optic flow motion (compensation) and negative values rotations in the opposite direction (errors). The response value for each animal is the sum of all the quarters of turns produced by the crab in the 3 min period. After that, the value is reported as the fraction of complete turns (360°) per min. Analyses were carried using R v3.4.4 (R Core Team, 2018) software. Data were analyzed using generalized linear mixed model (GLMM), accounting for both fixed and random effects. Due to the best fit of data under a normal distribution, LMM was used. LMM was generated using the lme4 package (Bates et al., 2015), setting both the seeing eye and optic-flow rotational direction as fixed factors and each individual as random effect. Significance of the parameters as well as their interaction was tested by likelihood ratio test. Outlier values were removed to satisfy normality assumption checked by analytical (Shapiro-Wilk) or by graphic methods. Specific a posteriori comparisons were performed, and *p*’s were adjusted by Bonferroni’s method. In a few cases (indicated in the text), paired *t*-tests were used. Significant differences were considered when *p*’s < 0.05. Data presented in the text and figures represent mean ± SEM.

## Results

### Unidirectionality in the Monocularly-Driven Optomotor Response of *Neohelice*

**Figure 2:**
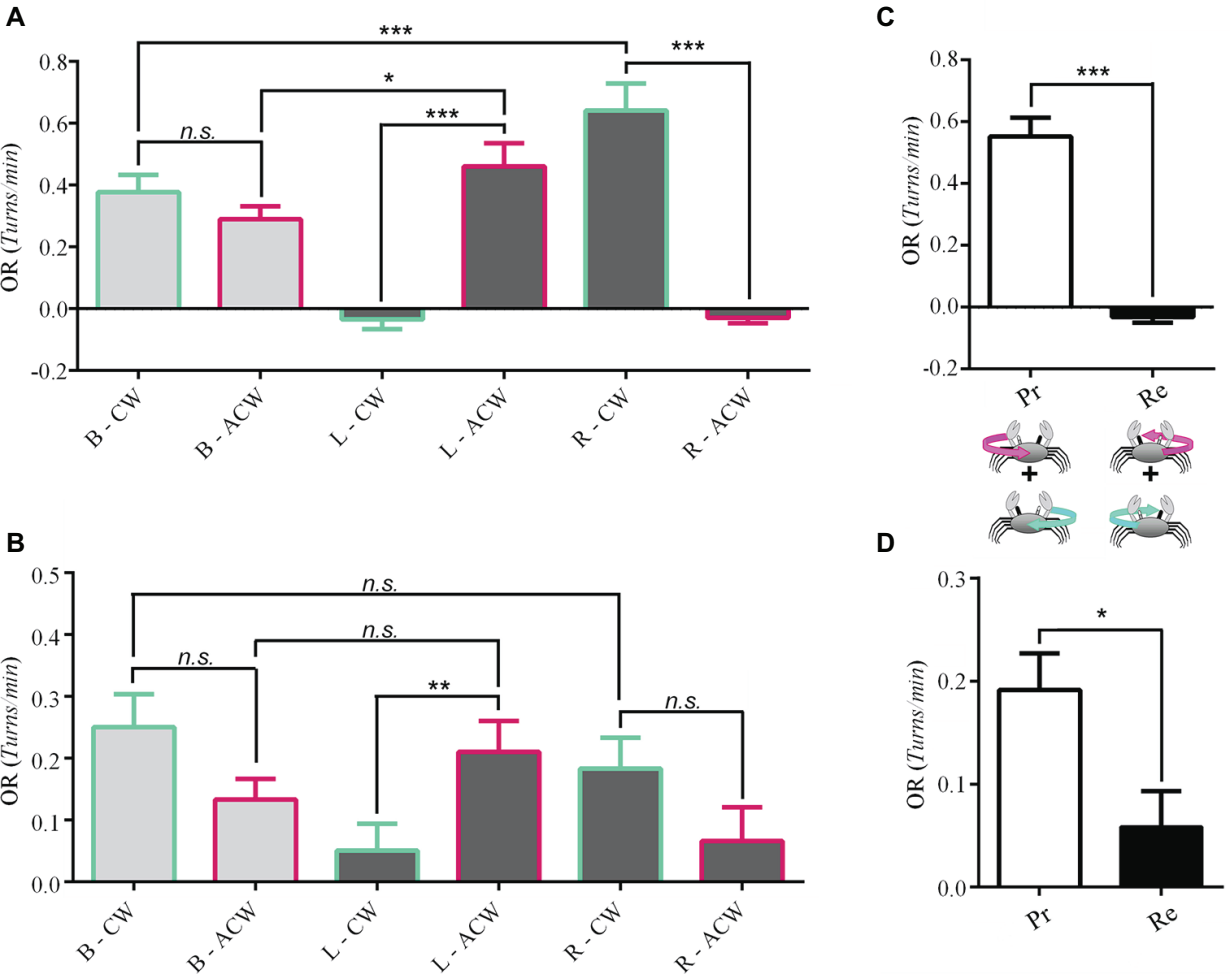
Unidirectional optomotor responses. The response of 19 *Neohelice* crabs and 27 *Uca* crabs were obtained under binocular or monocular visual conditions (left or right) to the clockwise (CW) and anticlockwise (ACW) optic flow directions. **(A)** In the binocular condition (B), there was no difference in the magnitude of response of *Neohelice* to both directions of optic flow. On the contrary, under the monocular conditions, they performed unidirectional OR. Seeing with the left eye alone (L), they only responded to ACW (*p* = 1.5 × 10^−10^), while seeing with the right eye alone (R) they only responded to CW motion (*p* = 2 × 10^−16^). LMM: seeing eye *x* direction: *χ*^2^ = 114.21, df = 2, *p* = 2 × 10^−16^; seeing eye: *χ*^2^ = 5.62, df = 2, *p* = 0.06; direction: *χ*^2^ = 3.69, df = 1, *p* = 0.05. **(B)** Similar results were observed in *Uca*, although for the right monocular condition, the tendency did not reach a significant effect (*p* = 0.055). LMM: seeing eye *x* direction: *χ*^2^ = 13.88, df = 2, *p* = 0.0009; seeing eye: *χ*^2^ = 3.75, df = 2, *p* = 0.15; direction: *χ*^2^ = 0.34, df = 1, *p* = 0.55. **(C)** A reanalysis of the data by pooling together (*n* = 35), the responses to progressive (Pr) or regressive (Re) optic flow (regardless whether it occurred over the left or the right eye) but considering the direction of motion observed by the lateral field of the viewing eye (crabs can see 360° but they have greater visual acuity at the lateral pole). Only the Pr motion (FTB) was effective (paired *t*-test, *t* = 8.9818, df = 34, *p* = 1.7 × 10^−10^). No response was obtained in the Re direction (BTF). **(D)** A similar result was obtained for *Uca* (*n* = 54, paired *t*-test, *t* = 2.5194, df = 46, *p* = 0.01529). *p < 0.05; **p < 0.01; ***p < 0.001.

We measured the OR of 19 *Neohelice* evoked by clockwise (CW) or anticlockwise (ACW) rotations of the visual panorama in animals seeing with the two eyes (binocular vision: B), with the left eye reversibly occluded (right monocular vision: R) and with the right eye reversibly occluded (left monocular vision: L). Results show that, under binocular conditions, the magnitude of the OR for the two directions of motion was not different (light gray bars in Figure 2A, *p* = 0.24). On the other hand, under monocular vision crabs displayed a highly directional OR. Interestingly, crabs seeing the stimuli with the left eye responded exclusively to anticlockwise motion (*p* = 1.5 × 10^−10^; Figure 2A). Conversely, under right monocular vision, the optic flow only evoked responses when the panorama moved clockwise (*p* = 2 × 10^−16^; Figure 2A).

Worthy of note, for both stimulus directions, the level of OR elicited in the preferred monocular condition was higher than in the binocular condition (R-CW vs. B-CW, *p* = 0.0004; L-ACW vs. B-ACW, *p* = 0.02).

### Unidirectionality in the Monocularly-Driven Optomotor Response of *Uca*

To investigate whether unidirectional optomotor response is particular of *Neohelice* or it is a general feature that extends to other crab species in different eubrachyuran families, we repeated these experiments using the fiddler crab *U. uruguayensis*, a member of the *Ocypodidae* family, which is sympatric with *Neohelice*. Fiddler crabs are named this way because males have a claw (major claw) that is much larger in size than the other one. For *U. uruguayensis*, it was reported an even proportion of right- and left-clawed crabs (Spivak et al., 1991).

Results from 27 crabs show that, similar to *Neohelice*, under binocular condition, the intensity of the OR was not different for the two directions of stimulation (light gray bars in Figure 2B, *p* = 0.08). Yet, when assessed in monocular conditions, fiddler crabs displayed directional OR preferences similar to those of *Neohelice*: crabs seeing the stimulus with the left eye responded mostly to anticlockwise motion (*p* = 0.006), while crabs seeing it with the right eye tended to respond mainly when the panorama moved clockwise (*p* = 0.055; Figure 2B). Although the latter contrast does not reach statistical significance, further analysis considering additional factors allowed to reveal a clear significant effect (see below). Unlike *Neohelice*, the preferred unidirectional monocular responses were not higher than in the binocular condition. Overall, the obtained levels of response in *Uca* were lower than in *Neohelice*. This is likely because the stimulus velocity and bar width were optimized for *Neohelice*.

### Progressive vs. Regressive Motion

Considering the monocular conditions in which animals responded more intensely to the panoramic motion stimulation (Figures 2A,B), we noticed that they all had in common that the lateral field of the viewing eye was being stimulated by FTB (progressive) motion. In fact, by pooling together the responses to progressive motion regardless the operating eye and comparing them with the corresponding data obtained for regressive motion in *Neohelice*, results are very clear. Crabs only respond to progressive motion (Figure 2C, paired *t*-test, *t* = 8.9818, df = 34, *p* = 1.7 × 10^−10^). The same result was obtained in *Uca* (Figure 2D, paired *t*-test, *t* = 2.5194, df = 46, *p* = 0.01529).

### Clawedness Affects Behavior in *Uca*

**Figure 3:**
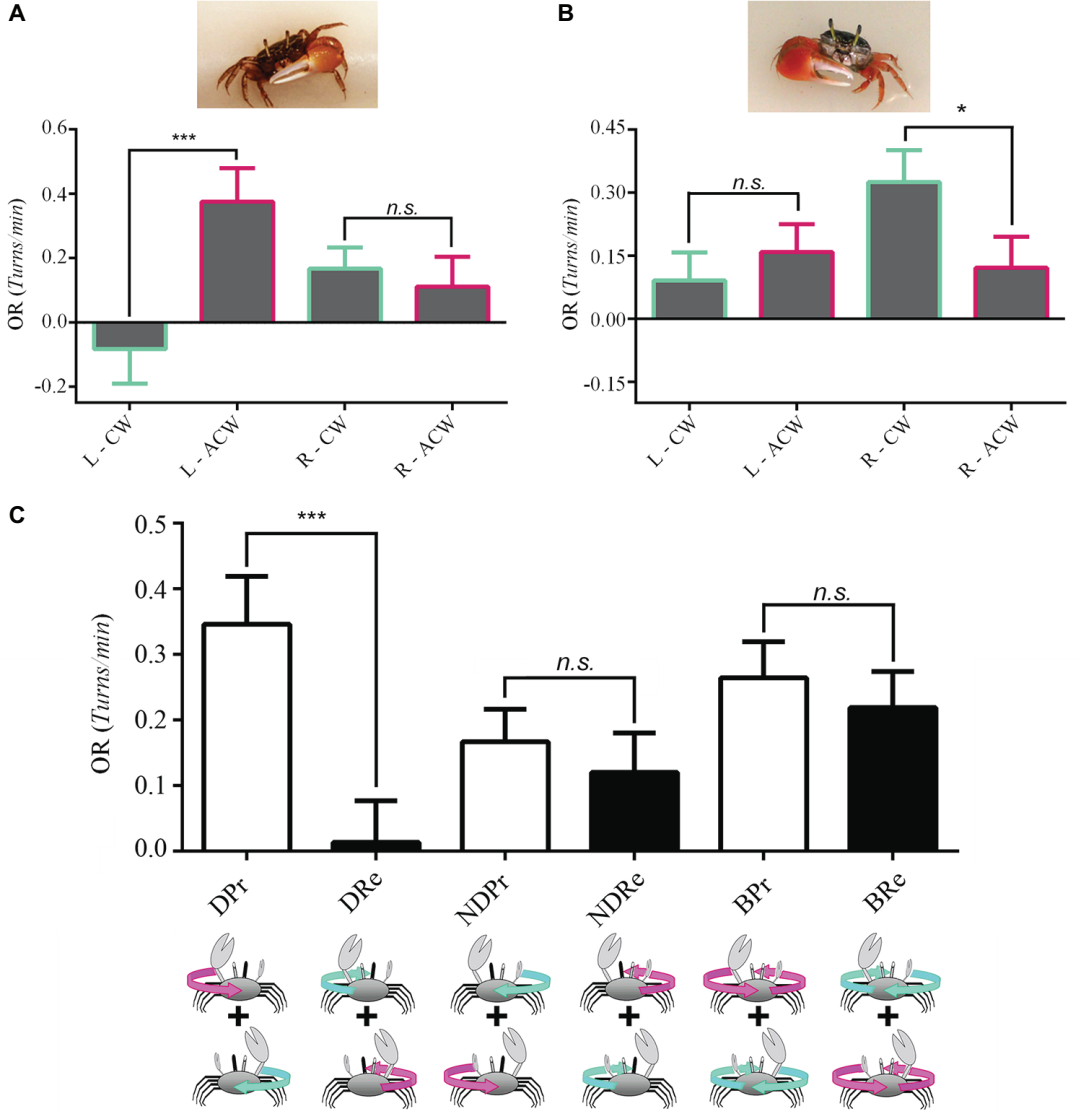
Eye dominance in *Uca* fiddler crabs. We made experiments separately in left-clawed [**(A)**, *n* = 15 crabs] and right clawed [**(B)**, *n* = 12] crabs. **(A)** Left-clawed animals only responded when seeing with the left eye to anticlockwise optic flow. No difference in the intensity of the OR between the two stimulus directions was obtained when animals perceived the optic flow with the right eye. LMM: seeing eye *x* direction: *χ*^2^ = 7.55, df = 1, *p* = 0.0006; seeing eye: *χ*^2^ = 0.0007, df = 1, *p* = 0.98; direction: *χ*^2^ = 4.43, df = 1, *p* = 0.035. **(B)** Right-clawed crabs only responded when the right eye was stimulated with a clockwise stimulus. No directional preference was observed for left eye stimulation. LMM: seeing eye *x* direction: *χ*^2^ = 5.49, df = 1, *p* = 0.019; seeing eye: *χ*^2^ = 2.62, df = 1, *p* = 0.11; direction: *χ*^2^ = 1.12, df = 1, *p* = 0.29. **(C)** Data regrouped considering the direction of optic flow with respect to the lateral part of the “dominant eye” (D, the one located on the same side of the major claw) or with respect to the non-dominant eye (ND). A significant difference between progressive (Pr) and regressive (Re) motion was obtained only for the dominant eye (*p* = 2.1 × 10^−5^). LMM: seeing eye and claw side combination: *χ*^2^ = 21.82, df = 5, *p* = 5.7 × 10^−4^. *p < 0.05; **p < 0.01; ***p < 0.001.

An animal is said to be lateralized if one side of its body is structurally or behaviorally different from the other (Byrne et al., 2004). In humans, handedness is the most studied aspect of brain asymmetries (Ocklenburg et al., 2013). Given that the presence of a major claw in *Uca* could potentially affect vision or generate behavioral differences, as was reported for amphipods (Munguia and Heldt, 2016), we performed experiments in 15 left- (Figure 3A) and 12 right-clawed (Figure 3B) crabs separately. With this separation, a remarkable lateralization of function became evident, since a complete unidirectional OR was only exhibited when the non-occluded eye corresponded with the major claw side. Interestingly, left-clawed *Uca* crabs only responded to anticlockwise optic flow, while right-clawed crabs only responded to a clockwise presentation of the optic flow (Figures 3A,B, respectively). In contrast, when seeing the stimulus monocularly with the eye corresponding to the minor claw side, responses were similar in the two directions of panoramic rotation (Figures 3A,B). In accordance, we regrouped the data considering the direction of optic flow with respect to the progressive (Pr) or regressive (Re) direction of motion perceived by the lateral part of the “dominant eye” (D, located at the same side of the major claw) or with respect to the direction of motion perceived by the lateral part of the non-dominant eye (ND). Pooling data from left- and right-clawed crabs together rendered a clear outcome stressing the asymmetry in the responses of the two sides and directions (Figure 3C). Notice that the first two bars (DPr, DRe, Figure 3C) now show a high significant difference, similar to that found for *Neohelice* (Figure 2C).

Surprisingly, although we might have expected that the extreme laterality found in monocular conditions would be reflected in the behavior of binocular crabs, they did not show a directional preference in the OR, responding similarly in the two directions of panoramic motion (Figure 3C, *p* = 0.124).

### Eye Dominance and Claw Size in *Neohelice*

Taking into account the results obtained in *Uca*, we reexamined a large series of results from previous pilot experiments performed in *Neohelice* aimed at establishing the best parameters of the optic flow for evoking the OR when tested monocularly. These experiments included changes in spatial frequency (0.035 vs. 0.058 cycles/degree), motion velocity (1 vs. 5 rpm) and timeframe to better assess the OR after eyes immobilization (4–5 vs. 6–8 days; results not shown). Despite the variety of test conditions, in all cases, monocular animals exposed to a progressive panoramic stimulus seen with the right eye consistently showed a slightly stronger OR than when they were exposed to the same progressive stimulus seen with the left eye (this tendency can be observed in the results of Figure 2A, by comparing R-CW and L-ACW responses). Pooling together results (*n* = 49) from animals tested in approximately the same day relative to the immobilization of the eyestalks (6–8 days) and with the same spatial frequency (0.035 cycles/degree) at 1RPM, we compared the magnitude of the OR driven by the right eye to clockwise rotations with that driven by the left eye to anticlockwise rotations (i.e., progressive motion direction for each eye). We found that the OR driven by the right eye was stronger, suggesting some level of eye dominance in this function (right eye seeing a CW stimulus: 0.65 ± 0.06 turns/min; left eye seeing an ACW stimulus: 0.48 ± 0.05 turns/min; paired *t*-test, *t* = 2.4315, df = 48, *p* = 0.01882).

Even though *Neohelice* males do not have a noticeable difference between the two claws, we wondered if the observed prevalence of the OR driven by the right eye could be related to a difference in claw size, as it was the case in *Uca*.

Because this was a *post-hoc* hypothesis derived from the analysis of our results, at the time of the experiment, there was no reason to measure the claws of the animals. Therefore, unfortunately, we do not have that information. However, because results from crabs derived from different capture efforts rendered always a similar outcome (see above), we measured the length of the right and left claw (including propodus and dactyl) in a population sample of 40 *Neohelice* adult male crabs. Interestingly, the right claw was slightly larger than the left one in 55% of the animals, while only in 20% of the cases the left one was slightly larger and in the rest there was no measurable difference. Furthermore, the mean value was significantly larger for the right claw compared with the left one (right: 23.39 ± 0.29 mm; left 23.09 ± 0.27 mm; paired *t*-test, *t* = 2.6902, df = 39, *p* = 0.01046). Therefore, although we were unable to establish a direct correspondence between the side preference of OR to progressive motion with the side of the larger claw in *Neohelice*, the sum of evidence in combination with what was observed in *Uca* support such a relation.

## Discussion

Results obtained here in two crab species are opposite to those reported in vertebrates (see Introduction) but very similar to those reported by Duistermars et al. (2012) in *Drosophila*: the monocular OR is maximal in the FTB (progressive) direction, binocular responses are less intense than the corresponding monocular FTB response, and the monocular BTF direction tends to produce “errors” where animals end up decompensating optic flow (see for example L-CW and R-ACW conditions in Figure 2A). These similarities between flies and crabs might, at first, appear strange given that monocular visual fields are very different in these two groups of arthropods. Flies possess monocular fields of view restricted to the ipsilateral field (about 150°) and only a small field of binocular superposition in the frontal part subtending about 20–30° (Beersma et al., 1977). In consequence, visual stimuli presented ipsilaterally in an area covering an angle between 15 and 165° are seen only by one eye. On the other side, crabs, with eyes mounted on the tip of movable stalks, have a complete visual field with each eye (Smolka and Hemmi, 2009; Astrada et al., 2012). That means that the area of binocular superposition encompasses 360°. Yet, the eyes are not uniform on their sensitivity. Nalbach and Nalbach (1987) demonstrated in several crab species that the most sensitive part of the eye in triggering OR is the lateral part of the ipsilateral field of view of each eye. Results presented here are consistent with those results and show that crabs use a subregion of their eyes, the lateral visual field, to optimally drive the OR.

The similarities found in the OR between flies and crabs indicate that the neural network underlying optic flow processing may share important commonalities in both systems. In flies, the neuropil involved in processing optic flow is the lobula plate (e.g., Heisenberg et al., 1978; Borst and Egelhaaf, 1989; Borst and Haag, 2002). Even though the center involved in optic flow analysis has yet to be identified in crustaceans, we have recently described in *Neohelice* a neuropil with many anatomical characteristics in common with the dipterous lobula plate (Bengochea et al., 2018).

Extensive studies have been made recording extracellularly from fibers of the optic tract in a variety of crustaceans including crabs, crayfishes, lobsters, etc. (Wiersma and Mill, 1965; Wiersma, 1966, 1970; Wiersma and Yamaguchi, 1967; Wiersma and Yanagisawa, 1971; Wiersma and York, 1972; Labhart and Wiersma, 1976). However, fibers with adequate properties to be involved in optomotor responses were not found. Wiersma and collaborators described a group of non-habituating “medium movement fibers” with optimal responses to medium velocities similar to those evoking the strongest optomotor responses and suggested a possible involvement in this behavior (Waterman et al., 1964; Wiersma, 1966) although no specific experiments toward this end were made. The only example of recorded fibers possibly associated with optomotor responses were made by Sandeman et al. (1975) in the crab *Carcinus maenas.* These fibers were unidirectionally sensitive to the movement of a striped drum around the animal and showed little habituation, but since the recordings were taken extracellularly, the morphological identification of the neurons was not feasible. Interestingly though, the authors describe their recording site as next to the lobula (medulla interna in the old terminology) and below the sinus gland (see Figure 2 in Sandeman et al., 1975), which coincides with the established location of the lobula plate of crabs (Bengochea et al., 2018). Based on these results and the ones presented here, we have just started a project aimed at intracellularly recording and staining tangential neurons from the lobula plate of *Neohelice* to investigate its role in optic flow processing.

In the present account, OR was measured through body rotations (fixed eyestalks). It could be argued that results may vary if measuring the OR through eye movements. However, we think this is unlikely because in the aforementioned study using *Drosophila* (Duistermars et al., 2012), both wing (which, as leg motion in crabs, results in body turns) and head movements (corresponding to eye movement in crabs) were measured, and parallel results were obtained in terms of preferred motion direction with the two outputs.

The characteristics of OR in monocular flies have been studied using tethered and free flying measurements and also analyzing walking trajectories (Wehrhahn and Hausen, 1980; Kern et al., 2000; Kern and Egelhaaf, 2000). Kern and Egelhaaf (2000) studied changes in the spontaneous trajectory of binocular and monocular flies (one eye covered with paint) in both free flying and walking blowflies and obtained similar results with the two locomotive methods: it was found that while binocular flies follow a straight trajectory, monocular flies turn slightly toward the seeing eye. These deviated trajectories were interpreted as compensatory movements made by the flies to reach optomotor equilibrium following the imbalance in the optic flow provoked by occluding one eye. Similar experiments in crabs may provide further support onto the similarities in the OR performance among invertebrates.

Considering vertebrates, the list of animals where monocular OR is more effectively elicited with BTF or regressive motion is wide. It includes rabbits, guinea pigs, prairie dogs, rhinoceros iguanas, gila monsters, leopard geckos, beaded lizards, black caimans, frogs, domesticated pigeons, and chickens (Mowrer, 1936; Fukuda, 1959; Tauber and Atkin, 1968; Collewijn, 1969; Jardon and Bonaventure, 1995). To explain unidirectionality, different interpretations have been suggested although none has proven infallible and covered all case reports (Fukuda, 1959; Tauber and Atkin, 1968). The stronger cases are related with the complete crossing of the visual pathways and with an afoveate type of vision (Fukuda, 1959; Tauber and Atkin, 1968). However, it is interesting that, at least in the case of frogs, the administration of certain drugs can affect the degree of asymmetry. For example, the injection of picrotoxin (a GABAergic antagonist) or piribedil (a D2 dopamine agonist but with known muscarinic receptors interactions) erases all traces of asymmetries in the OR between the two directions of the panoramic stimulus, meaning that the asymmetry is not constrained by anatomical connections but based on complex neurotransmitter-mediated communication between the two eyes (Yücel et al., 1990; Jardon and Bonaventure, 1995).

Our results in two species of crabs, in coincidence with those previously obtained in flies, strengthen the notion of an OR preference for progressive motion among invertebrates. Whether the difference in the preferred direction of monocular OR disclosed between vertebrates and arthropods relates to particular evolutionary traits (related to the organization of the circuitry underlying optic flow analysis) or to the type of optomotor responses performed by the particular animal under study is not clear. Flies do not share with the rest of the evaluated vertebrate species the ability of moving the eyes. Instead, they move the whole head and body to scrutinize the images. Crabs, on the other hand, perform eye movement in the OR similarly to vertebrates (Land, 1999). Yet, they share the preferred direction with flies and not with vertebrates. Although not directly aimed to investigate this issue, a recent study from Daly et al. (2017) also shows a preference for FTB motion in stomatopods, a singular type of crustacean that shows independent movement of their left and right eyes. Therefore, the effective direction of unilateral OR does not seem to be related with the type of compensatory movements displayed by the animal. Rather, the opposite directional preferences between vertebrates and invertebrates seem to be related to a different ground plan in the circuitry underlying optic flow analysis of these traits. In particular, the preferred direction could depend on the type of interocular connections or on the response preference of the directional neurons responding to optic flow present in each system. In this line of thought, it is known from studies in the lobula plate that the HS tangential cells that are sensitive to horizontal optic flow respond only to FTB optic flow (Hausen, 1982a,b). Thus, the sensitivity of HS cells could explain by itself the behavioral response in monocular conditions. In the present series of experiments, we evaluated two crab species belonging to different families within Brachyura. The general results obtained in *Neohelice* and *Uca* were similar since both had a FTB preferred unidirectional optomotor response. However, we found a big difference regarding eye dominance in this task. In *Uca*, a strong response to FTB optic flow was obtained only when OR was driven by the eye corresponding to the side of the major claw. When seeing with the eye contralateral to the major claw, a reduced response was obtained for the two directions. Surprisingly, no difference was observed between the responses elicited by the two motion directions in binocular conditions.

What might be the role in *Uca* for an extreme laterality in the OR is a question that remains open. One possibility is that, on the side of the larger claw (which is used for courtship and defense), the animal possesses a greater visual sensitivity that prompts them to turn around more easily toward this side. Alternatively, animals could have similar visual sensitivity but an asymmetric ability to rotate, being more prone to do it toward the side of the major claw. Eye dominance could also be associated to the asymmetries present in the two sides of the body in fiddler crabs. After maturation, when asymmetries begin, the handiness of a fiddler crab is conserved after removal of the major claw meaning that physiological or nervous asymmetries are present (Ahmed, 1978; Mariappan et al., 2000). Under these conditions, an overdevelopment of brain circuits has been reported (Young and Govind, 1983), but no data are available from the optic lobes.

Interestingly, despite the difference between its claws is small, *Neohelice* also appears to have a degree of lateralization of the OR. This is suggested by the fact that a stronger OR was observed on animals seeing the optic flow with the right eye. Although we did not measured the claws of the animals evaluated in that experiments, a later measurement on a group derived from a different capture effort showed that indeed most *Neohelice* crabs have the right claw slightly larger than the left one.

Left-right asymmetries in the nervous system or behavior of several invertebrate species including mollusks, insects, and nematodes have been described (see Frasnelli, 2013 for a review). For example, octopuses display lateralized eye use in observing and attacking prays (Byrne et al., 2004), bees present lateralization of function in olfactory and visual learning (Letzkus et al., 2006, 2008) and bumblebees tend to rotate always in the same direction around each inflorescence (Kells and Goulson, 2001). The present study was not aimed to investigate lateralization. Yet, the results obtained on this subject were clear-cut, especially in *Uca*. Further experiments would be addressed to evaluate in higher depth lateralization in crabs and to know whether the eye dominance is specific for the OR or if it prevails in other visually guided behaviors, such as the escape response to visual threats.

## Contribution to the Field

All animals stabilize panoramic optic flow through compensatory movements of the eyes, the head or the whole body, a behavior known as optomotor response (OR). Under monocular vision, some animals, including various species of mammals, birds, reptiles, amphibious, fishes, and flies, show a unique effective direction of compensatory motion (unidirectional OR). In vertebrates, OR operates only when the panorama moves in a back to front direction, whereas in the fly, so far the only invertebrate that has been tested, it occurs only when the panorama moves in the opposite direction. In order to disclose whether the front to back directional preference of the fly, OR was an exception or a trend that distinguishes invertebrates from vertebrates, we analyzed the directional preference in two species of crabs belonging to different families (*Varunidae* and *Ocypodidae*) within Brachyura. We found that the two species have the same unidirectional preference of OR than the fly. Our results provide strong support for a commonality in the circuit underlying OR among arthropods. Additionally, we show that in fiddler crabs, there is remarkable eye dominance for OR associated to the size of the larger claw.

## Data Availability

The datasets generated for this study are available on request to the corresponding author.

## Ethics Statement

Research was conducted in accordance with the Ethical Reference Frame for Biomedical Investigations of the Consejo Nacional de Investigaciones Científicas y Técnicas de Argentina, equivalent to the standard procedures for animal care and use of the US NIH.

## Author Contributions

All the authors had full access to all the data in the study and take responsibility for the integrity of the data and the accuracy of the data analysis. JS and DT helped in study concept and design. YB helped in acquisition of data. YB, JS, and DT helped in analysis and interpretation of data. JS helped in drafting of the article. YB, JS, and DT helped in critical revision of the article. JS and DT obtained funding. JS and DT helped in study supervision.

### Conflict of Interest Statement

The authors declare that the research was conducted in the absence of any commercial or financial relationships that could be construed as a potential conflict of interest.
